# Supplementary material for: WordCommentsAnalyzer: A windows software tool for qualitative research
Source: F1000Res. 2018 Sep 4;7:536. Originally published 2018 May 3. [Version 2] doi: 10.12688/f1000research.14819.2 (PMC6137411; doi:10.12688/f1000research.14819.2)
Supplement: Supplementary file 3 [file f1000research-7-17614-s0002.tgz › b2a79496-4ada-47c4-9682-a99ce867836d.zip!/Eight Iranian Health Journals/Yafteh _19.docx]

© Yafteh  . This work is licensed under CC BY 4.0 (https://creativecommons.org/licenses/by/4.0/).

Volume & Issue: دوره 15 - شماره 4

Title: ارتباط KIRهای فعال‌کنندگی با استعداد ابتلا به سل در جمعیت لر

http://yafte.lums.ac.ir/article-1-1439-fa.html

مقدمه: سلول‌های کشنده طبیعی (NK)،از طریق مکانیسم‌هایی همچون سایتوتوکسیسیتی و تولید سایتوکاین‌،‌ اولین خط دفاعی علیه عفونت‌ها بشمار می‌آیند. توانایی سایتوتوکسیسیتی سلول‌های NK‌ با پذیرنده‌های شبه ایمونوگلوبولینی سلول‌های کشنده (KIR) موجود در سطح سلول ارتباط دارد. برهمکنش میان KIRها‌ و مولکول‌های آنتی‌ژن لکوسیتی انسان (HLA) کلاس I، پاسخ‌های سلول‌های NK را در حذف سلول‌های عفونی‌ تنظیم می‌کند. بنابراین، در ادامه یک مطالعه مقدماتی،هدف این مطالعه تکمیلی بررسی تأثیر ژن‌های KIR، ژن‌های لیگاند HLA و ترکیب KIR-HLA در استعداد ابتلا به سل (TB)‌ در جمعیت لر بود. بحث و نتیجه‌گیری: این یافته‌ها دلالت بر عدم توازن ژنتیکی بین ژن‌های KIR مهاری و فعال‌کنندگی و ترکیبات KIR-HLA در بیماران سلی جمعیت لر دارند. سطح پایین KIRهای فعال‌کنندگی و به ویژه KIR3DS1 و ترکیب آن با HLA-B Bw4Ile80 ممکن است در استعداد ابتلا به سل در جمعیت لر تأثیر داشته باشد. در واقع، این یافته‌ها نتایج مطالعه مقدماتی ما را تأیید کرد. یافته‌ها: در این مطالعه، فراوانی KIR3DS1 به طور معنی‌داری در گروه کنترل از گروه بیمار بالاتر بود(45٪ در مقابل 24٪، 0204/0=Pc). همچنین، ترکیب KIR3DS1+HLA-B Bw4Ile80در افراد کنترل در مقایسه با بیماران سلی فراوان‌تر بود(25٪ در مقابل 4٪، 0034/0=Pc). مواد و روش‌ها: DNA ژنومی 50 بیمار سلی از استان لرستان برای 16 ژن KIR‌ و 5 لیگاند HLA کلاس I‌ اصلی آنها، به روش واکنش زنجیره‌ای پلیمراز با استفاده از پرایمرهای اختصاصی توالی (PCR-SSP) ژنوتایپ شدند. درنهایت، این نتایج با نتایج 100 فرد لر سالم مقایسه شدند.

Title: فراوانی عوارض مزمن دیابت نوع دو در بیماران کاندید مصرف انسولین به علت عدم کنترل قند خون با داروهای خوراکی

http://yafte.lums.ac.ir/article-1-1440-fa.html

مقدمه: دیابت نوعی بیماری مزمن و شایع در سرتاسر جهان است. این بیماری دارای عوارض مزمن جسمی زیادی است. هدف از انجام این مطالعه، بررسی عوارض جسمی در بیماران دیابتی نوع دو بود که به علت عدم کنترل قند خون با داروهای خوراکی در بیمارستان شهدای عشایر خرم آباد بستری شده بودند.   بحث و نتیجه­گیری: با توجه به شیوع بالای عوارض قلبی عروقی در بیماران و عدم وجود برنامه مشخصی برای ارزیابی این عوارض، توصیه می ­ گردد که با تدوین برنامه   های ارزیابی دوره   ای وضعیت قلبی عروقی، از بروز این عوارض پیشگیری گردد.   یافته­ها: تعداد بیمارانی مورد بررسی 299 نفر بود و از این تعداد 177 نفر زن بودند. میانگین سنی بیماران 7/14±6/58 سال بود. شایعترین عارضه، بیماری های قلبی (5/41٪) و پس از آن، رتینوپاتی (4/37%) بود. بین وجود رتینوپاتی و نفروپاتی و مدت زمان سپری شدن از شروع بیماری ارتباط معنی داری وجود داشت.   مواد و روش­ها: این مطالعه توصیفی مقطعی گذشته نگر بر روی تمام بیماران دیابتی نوع دو که به علت عدم کنترل قند خون با داروهای خوراکی، برای درمان با انسولین در سال های 1388 و 1389 بستری شده انجام گردید. اطلاعات بیماران با استفاده از پرسشنامه از پرونده های بیماران جمع آوری شدند و با استفاده از نرم افزار آماری و آزمون آماری مجذور کای و دقیق فیشر تجزیه و تحلیل شدند. مقدار احتمال کمتر از 05/0 معنی دار در نظر گرفته شد.

Title: اثربخشی درمان شناختی مبتنی بر حضور ذهن در کاهش اضطراب بیماران کرونر قلبی: با پیگیری دو ماهه

http://yafte.lums.ac.ir/article-1-1441-fa.html

مقدمه: از مسائل مهمی که بیماران قلبی با آن روبرو هستند مشکلات روحی و روانی همچون اضطراب می باشد، که تقریباً 65 درصد بیماران آن را تجربه می کنند و به همین سبب موجب تأخیر در بازگشت به کار، کاهش کیفیت زندگی و افزایش خطر مرگ می شود. هدف از این پژوهش تعیین و مقایسه میانگین اضطراب مبتلایان قبل، بعد و دو ماه بعد از مداخله در دو گروه (کنترل و آزمایش) بود. بحث و نتیجه‌گیری: با توجه به تأثیر درمان شناختی مبتنی بر حضور ذهن بر میزان اضطراب مبتلایان و تداوم اثر آن، به کارگیری این روش در همه‌ سطوح پیشگیری و درمان مبتلایان به بیماریهای جسمی ضروری بنظر می رسد. یافته‌ها: با توجه به یافته ها ملاحظه شد که اضطراب مبتلایان بعد و دو ماه بعد از مداخله کاهش قابل توجهی در مقایسه با قبل از مداخله داشته است و با سطح 01/0< P در گروه آزمایش تفاوت آماری معنی داری مشاهده گردید. مواد و روش‌ها: این پژوهش، مطالعه ای آزمایشی، دو گروهی و سه مرحله ای است که بر روی 40 مرد مبتلا به بیماری کرونر قلب که به طور تصادفی در دو گروه آزمایش و کنترل قرار گرفتند انجام شد. ابزار گردآوری داده ها پرسشنامه‌ اضطراب بک بود و به منظور دستیابی به نتایج از نرم افزار19 SPSS و تحلیل کوواریانس استفاده شد. شرکت کنندگان در این پژوهش قبل، بعد و در دوره پیگیری مداخله به کمک پرسشنامه‌ اضطراب بک مورد بررسی قرار گرفتند.

Title: بررسی ارتباط سرطان های کولورکتال با سابقه فامیلی سرطان های دستگاه گوارش و سابقه فردی پولیپ های روده بزرگ در شهر خرم آباد در سال1391

http://yafte.lums.ac.ir/article-1-1442-fa.html

مقدمه: هدف از این مطالعه بررسی ارتباط سرطان‌های کولورکتال با سابقه فامیلی سرطان‌های دستگاه گوارش و سابقه فردی پولیپ‌های روده بزرگ در شهر خرم‌آباد بود. مواد و روش‌ها: در این مطالعه مقطعی در طی سال 1391، تعداد 50 بیمار با تشخیص قطعی سرطان روده بزرگ بر اساس کولونوسکوپی و تایید پاتولوژی و 56 فرد همسان شده به لحاظ سنی و جنسی با بیماران، از بیماران سرپایی مراجعه کننده به درمانگاه‌های پوست و چشم بیمارستان شهدای عشایر به شرط نداشتن بیماری‌های گوارشی به‌عنوان گروه کنترل، از نظر سابقه خانوادگی سرطان‌های دستگاه گوارش در بستگان درجه اول و نیز سابقه ابتلا به پولیپ کولورکتال توسط یک پرسشنامه خودساخته مورد بررسی قرار گرفتند. از آزمون‌ دقیق فیشر و نیز محاسبه نسبت شانس جهت تجزیه و تحلیل اطلاعات استفاده شد. بحث و نتیجه‌گیری: نتایج مطالعه حاضر باید در آینده با مطالعات بیشتر و نمونه‌گیری در حجم بزرگتر تایید شود تا با تدوین برنامه‌های جامع پیشگیری بر مبنای غربالگری، زمینه مبارزه هر چه جدی‌تر با این سرطان فراهم گردد. یافته‌ها: میانگین سنی بیماران 5/15±8/52 سال بود و 56% بیماران مرد بودند. بین سابقه سرطان‌های معده و روده بزرگ در بستگان درجه اول با بروز سرطان کولورکتال در خود فرد ارتباط آماری معناداری دیده شد (05/0P<). نسبت شانس ایجاد سرطان کولورکتال در افراد با سابقه مثبت سرطان معده و سرطان روده بزرگ در بستگان درجه اول، به ترتیب 96/3 (61/6-44/1CI= ) و 75/6 (1/11-4/2CI=) برابر افراد گروه کنترل بود . بین سابقه سرطان مری در بستگان درجه اول با بروز سرطان روده بزرگ در خود فرد ارتباط آماری معناداری دیده نشد. همچنین ارتباط معناداری بین سابقه فردی ابتلا به پولیپ‌های روده بزرگ با بروز سرطان کولورکتال دیده شد (004/0P= ).

Title: بررسی سرواپیدمیولوژیک بیماری هپاتیت ب در شهرستان‌های کوهدشت و سپیددشت در سال 1391

http://yafte.lums.ac.ir/article-1-1443-fa.html

مقدمه: هپایت یک بیماری است که کبد را متورم کرده و فعالیت آن را مختل می سازد. اغلب از راه تزریق خون آلوده و فراورده های خونی آلوده منتقل می‌شود . این بیماری ممکن است مزمن شود و علائمی شبیه آنفلوانزا داشته باشد. خستگی، ازدست دادن اشتها، درد عضلات و مفاصل، تهوع و استفراغ از علائم بارز آن می باشد . هدف ازاین مطالعه بررسی میزان شیوع هپاتیت ب درجمعیت روستایی مناطق گراب ، چغابل ، پادروند و تنگ پنج بر حسب سن، جنس، شغل..... ومقایسه آن با میزان شیوع در سایر مناطق مشابه استان وکشورمی باشد. مواد و روش‌ها: با هدف تعیین شیوع سرمی عفونت HbsAg در مناطق گراب، پادروند، چغابل و تنگ پنج و تعیین رابطه میان عوامل شناخته شده اپیدمیولوژیک با عفونت، از جمعیت‌های این مناطق پس از تکمیل پرسشنامه ای که علائم بیماری هپاتیت ب در آن لحاظ شده بود نمونه خون گرفته شده و با روش الیزا مورد آزمایش قرار گرفتند . بحث و نتیجه‌گیری: برمبنای نتایج بدست آمده از این مطالعه شیوع هپاتیت ب در مناطق یاد شده به مراتب بیش از میانگین کشوری( 2 تا 3 در صد) بوده که با داده های نظام مراقبت بیماری در مناطق یادشده همخوانی دارد . اگرچه یافته های مطالعه الگوی اصلی انتقال بیماری را تعیین نمی کند ولی با توجه به ترکیب سنی و جنسی افراد مبتلا ( نسبت تقریباٌ برابر در مرد وزن و فزونی ابتلاء در افراد متأهل ) می توان ادعا کرد که الگو تاحدی زیاد به نفع انتقال جنسی است . ضمن اینکه کلیه گروههای سنی وجنسی درمعرض خطرآلودگی هستند اما از نظرجنس مردان(9/51 %) و از نظرسنی گروههای سنی بین 20-40 ساله بیشترین موارد ابتلا راداشته اند. شغل و سابقه تزریق خون ، بعد خانوار وسابقه واکسیناسیون دارای رابطه معنی داری با عفونت HbsAg بودند . یافته‌ها: از 454 فرد مورد مطالعه 227 نفر مرد و 227 نفر زن بودند ( نسبت جنسی برابر ) که از این عده 27 نفر ( 2/6 درصد ) از نظر بیماری هپاتیت ب آزمایش الیزا مثبت داشتند .درمیان افراد مبتلا 14 نفر مرد ( 9/51 در صد ) و 13 نفر ( 1/48 در صد ) زن بودند . بیشتر موارد مثبت بین گروههای سنی20 تا 40 سال بودند. گروههای شغلی خانه دار ، راننده و دانشجو بیشترین در صد ابتلا را داشتند .

Title: بررسی اثر آلودگی صوتی بر هورمون های محور هیپوفیز-گناد در موش صحرایی نر

http://yafte.lums.ac.ir/article-1-1447-fa.html

مقدمه: سرو صدا نوعی از آلودگی محیطی است که کیفیت زندگی انسان ها را تحت تأثیر قرار می دهد. تحقیقات زیادی روی تأثیر استرس صوتی بر قسمت های مختلف بدن انجام شده ولی تا به حال مطالعات اندکی در زمینه تأثیر این استرس روی سیستم جنسی صورت گرفته است. به همین دلیل تصمیم گرفته شد تا به بررسی اثر آلودگی صوتی بر میزان هورمون های تستوسترون، LH و FSH پرداخته شود. مواد و روش‌ها: 2 گروه 10تایی موش صحرایی نر در 2 محیط تجربی و معمولی به مدت 50 روز قرار داده شدند که در محیط تجربی صوتی با شدت 120-90 دسی بل و فرکانس 350 - 300 هرتز توسط دستگاهی روزانه از ساعت 7 شب تا 7 صبح پخش می شد. بعد از گذشت 50 روز و با استفاده از روش خونگیری از دم، میزان هورمون های تستوسترون، LH و FSH در سرم خون با استفاده از روش الایزا سنجیده شد و نتایج توسط آزمون t با درجه معنی داری 05/0P< بررسی شدند. یافته‌ها: مطالعات آماری نشان داد که میانگین ترشح هورمون های تستوسترون، LH و FSH در موش های صحرایی نر تحت صوت در مقایسه با گروه کنترل اختلاف معنی داری داشته است (05/0P<). بحث و نتیجه‌گیری: در مجموع می توان گفت که استرس های صوتی باعث کاهش میزان هورمون های جنسی می شوند.

Title: بررسی مقاومت سودوموناس آئروژینوزا به داروی سفتازیدیم وایمیپنم در بیمارستان امام رضا (ع) کرمانشاه طی سالهای 89-85

http://yafte.lums.ac.ir/article-1-1448-fa.html

مقدمه: سودوموناس آئروژینوزا از مهم ترین عوامل ایجاد عفونت های بیمارستانی می باشد که می تواند در بیماران با ضعف سیستم ایمنی گستره ای از عفونت ها را ایجاد نماید. لذا وضعیت مقاومت آنتی بیوتیکی این ارگانیسم مهم را نسبت به دو داروی سفتازیدیم و ایمیپنم به عنوان داروهای مهم و شایع در درمان سودوموناس آئروژینوزا بررسی کردیم. مواد و روش‌ها: کلیه نمونه های خون، ادرار، مایعات و... ارسالی جهت کشت به آزمایشگاه مرکز آموزشی درمانی امام رضا (ع). وابسته به دانشگاه علوم پزشکی کرمانشاه طی مدت 5 سال (89-85)، پس از کشت در محیط های مکانگی آگار ، بلاد آگار و محـیط‌های کشت افتراقی و تشخیص باکتریهای سودوموناس آنتی بیوگرام شدند. الگوی آنتی بیوگرام این باکتری با روش انتشار دیسکKirby-Bauer و هاله عدم رشد آن طبق استاندارد های کمیته ملی برای آزمایشگاه های بالینی NCCLS)) مورد بررسی قرار گرفت. یافته‌ها: طی5 سال در این مرکز تعداد 364 مورد سودوموناس از نمونه های ارسالی جدا سازی شد که بیشترین شیوع در نمونه های ادراری با 5/51% بود. نتایج بررسی آنتی بیوتیکی نشان می دهد این ارگانیسم نسبت به سفتازیدیم و ایمیپنم به ترتیب56% و 7/24% مقاومت دارد و میزان مقاومت روند افزایشی سالانه را نشان می دهد. بحث و نتیجه‌گیری: در این مطالعه مشاهده گردید میزان مقاومت به دو آنتی بیوتیک سفتازیدیم و ایمیپنم طی پنج سال گذشته افزایش یافته که با مطالعات مختلف صورت گرفته در کشور های منطقه و همجوار هماهنگی دارد. اما با مطالعات کشور های صنعتی اختلاف واضحی را نشان می دهد. این مسئله بیان کننده ی اهمیت استفاده صحیح از این آنتی بیوتیکها در کشور های در حال توسعه است .

Title: بررسی ارتباط سبک‏های تنظیم شناختی هیجان با تکانشگری و اختلال شخصیت مرزی در دانشجویان

http://yafte.lums.ac.ir/article-1-1452-fa.html

مقدمه: تنظیم شناختی هیجان نقش برجسته ای در کنترل اختلالات روانی دارد. هدف مطالعه حاضر بررسی رابطه سبکهای تنظیم شناختی هیجان با صفات مرزی و رفتارهای تکانشی دانشجویان بود. مواد و روش‌ها: نمونه آماری این تحقیق شامل 200 نفر از دانشجویان دانشگاه پیام نور بودند. برای جمع آوری داده‌ها از پرسشنامه‌های صفات مرزی، رفتارهای تکانشی بارت و تنظیم شناختی هیجان استفاده شد. سپس داده‌ها با استفاده از آزمون ضریب همبستگی پیرسون و تحلیل رگرسیون چندگانه مورد تحلیل قرار گرفتند. بحث و نتیجه‌گیری: آموزش راهبردهای مؤثر تنظیم شناختی هیجان بهتر است به عنوان بخشی از مداخله در درمان رفتارهای تکانشی و اختلال شخصیت مرزی مورد نظر قرار گرفته شود. یافته‌ها: بین متغیر اختلال شخصیت مرزی با سبک‌های سرزنش خود، نشخوار فکری، فاجعه‌آمیز بودن، سرزنش دیگران تنظیم شناختی هیجان رابطه مثبت، ولی با سبک‌های پذیرش و ارزیابی مجدد مثبت رابطه منفی وجود داشت (5 0/0 >P). بین متغیر تکانشگری با سبک‌های سرزنش خود، نشخوار فکری، فاجعه آمیز بودن، سرزنش دیگران تنظیم شناختی هیجان رابطه مثبت، ولی با سبک‌های پذیرش و ارزیابی مجدد مثبت ارتباط منفی وجود داشت (5 0/0 > P). نتایج ضریب همبستگی چند متغیری به روش Enter نشان داد که سبکهای تنظیم شناختی هیجان در تبیین متغیر اختلال شخصیت مرزی (646/0 ) و تکانشگری (645/0 ) سهم دارند.

Title: بررسی سطح سرمی ویتامین D در مبتلایان به صرع تحت درمان با دو گروه داروهای القاء کننده و غیر القاء کننده آنزیمی

http://yafte.lums.ac.ir/article-1-1453-fa.html

مقدمه: در بیماران مصرف کننده داروهای ضد تشنج، تغییرات ویتامین D و مینرال های سرم گزارش شده است. این مطالعه جهت مقایسه تغییرات ویتامین D و مینرال های سرم دربیماران مصرف کننده دو گروه دارویی داروهای ضد تشنج (القاء کننده و غیر القاء کننده آنزیمی) طراحی شده است. بحث و نتیجه‌گیری: در بیماران تحت درمان با داروهای القاء کننده آنزیمی کمبود ویتامین D شایع تربوده اما فراوانی هیپرپاراتیروئیدیسم ثانویه کم تر است. یافته‌ها: میانگین ویتامین D سرم در گروه القا کننده کم تر از گروه غیر القا کننده بود (به ترتیب3/8± 9/15 در مقابل 8/14±2/24 02/0= (P.فراوانی کمبود ویتامین D در گروه داروهای القاء کننده 84% و در گروه داروهای غیر القاء کننده 48% بود (016/0=. (P میانگین کلسیم سرم در گروه داروهای القاء کننده به طور معنی دار کم تر از گروه غیر القاء کننده بود (به ترتیب 2/0±7/8 در مقابل 7/0±0/9 ،05/0=. ( P هیپرپاراتیروئیدیسم ثانویه در4% گروه داروهای القاء کننده و 24% گروه غیر القاء کننده وجود داشت (016/0=. (P مواد و روش‌ها: در این مطالعه 22 بیمار تحت درمان با داروهای القاء کننده آنزیمی با 25 بیمار داروهای غیر القاء کننده مقایسه شدند.بیماران دو گروه از نظر سن ،جنسیت و اندکس توده بدنی همانند سازی شدند. میزان کلسیم، فسفات، پاراتورمون و 25 هیدروکسی ویتامین D در دو گروه اندازه گیری شد. اندازه گیری کلسیم با روش کالریمتری و اندازه گیری پاراتورمون و ویتامین D با روش الیزا انجام شد.

Title: شناسایی گونه‌های آسپرژیلوسی با کاربرد تک آنزیم محدودساز MwoI در روش RFLP مبتنی بر آمپلیفیکاسیون ناحیه ژنی rDNA

http://yafte.lums.ac.ir/article-1-1454-fa.html

مقدمه: گونه‌های آسپرژیلوس بعضاً با بیماریهای انسان مرتبط هستند. یافتن یک روش دقیق و سریع برای شناسایی این قارچها اهمیت ویژه-ای دارد و می‌تواند در غربالگری بیماری و اهداف اپیدمیولوژیک مؤثر باشد. بحث و نتیجه‌گیری: روش PCR-RFLP مبتنی بر ژن DNA ریبوزومی، برای تعیین گونه‌های آسپرژیلوسی مورد استفاده قرار گرفت. کاربرد آنزیم محدودساز MwoIما را قادر نمود که بیشترین افتراق را در بین گونه‌های آسپرژیلوسی ایجاد نماییم و گونه‌های مهم آسپرژیلوسی را در مدت زمان کوتاهی شناسایی کنیم. یافته‌ها: ما موفق به شناسایی هشت گونه مهم آسپرژیلوس شامل: آسپرژیلوس فلاووس، آسپرژیلوس نایجر، آسپرژیلوس فومیگاتوس، آسپرژیلوس ترئوس، آسپرژیلوس کلاواتوس، آسپرژیلوس اکراسئوس، آسپرژیلوس آمستلوئیدامی و آسپرژیلوس نیدولانس با کاربرد روش مولکولی آزمون شدیم. شناسایی و افتراق مهمترین گونه‌های آسپرژیلوس بدست آمده از کشت کوتاه مدت 36 ساعته و استفاده از پروفایل معرفی شده شامل استخراج DNA، PCR و هضم آنزیمی برای تقریباً تمام نمونه‌های آزمون شده زمانی در حدود یک روز کاری نیاز داشت. مواد و روش‌ها: نمونه‌های ما شامل گونه‌های مختلف آسپرژیلوسی از نمونه‌های بالینی و محیطی چهار بیمارستان آموزشی بودند. آسپرژیلوس‌ها ابتدا با روش مورفولوژیک شناسایی شدند. توده‌های میسلیومی آسپرژیلوسی از کشت‌های سابورود براث استخراج شده برای استخراجDNA به روش فنل کلروفرم- گلاسبید مورد استفاده قرار گرفتند. آمپلیفیکاسیون ژن rDNA با استفاده از پرایمر‌های یونیورسال ناحیه ITS انجام شد و محصولات واکنش زنجیره‌ای قابل هضم با آنزیم محدودساز MwoI با استفاده از روش PCR- RFLP مورد تفکیک قرار گرفتند.

Title: اثر زهر زنبور عسل برروی قدرت تمایزی-D آلفا توکوفرول سوکسینات (ویتامین E) در رده سلول سرطانی حاد پرومیلوسیت HL-60

http://yafte.lums.ac.ir/article-1-1455-fa.html

مقدمه: لوسمی حاد پرومیلوسیت بدخیم ترین نوع لوسمی حاد است که با جابجایی کروموزومی (15:17) و توقف سلول‌ها در مرحله‌ پرومیلوسیت مشخص می شود. امروزه از روش تمایز درمانی جهت درمان سرطان استفاده می‌شود. مطالعات قبلی نشان داده‌اندکه ویتامین E سبب مهار تکثیر و القاء تمایز سلول‌های HL-60 به سمت مونوسیت می‌شود. با توجه به اینکه استفاده از غلظت‌های بالای ویتامین E دارای اثرات جانبی بوده و استفاده از این غلظت‌ها جهت القاء تمایز امکان پذیر نیست، لذا سعی می‌شود با استفاده از ترکیبات که دارای خاصیت ضد تکثیری بر روی سلول‌ها هستند، توان تمایزی آن را افزایش داد. از آنجایی‌که اثرات ضد تکثیری زهر زنبور عسل بر روی رده‌های سلولی گوناگون اثبات شده است، در این مطالعه اثر آن برعملکرد تمایزی ویتامین E بررسی شد. مواد و روش‌ها: در این بررسی از ایمونوسیتوشیمی، تست احیاءNBT و رنگ آمیزی رایت -گیمسا جهت بررسی تمایز سلولی استفاده شد. جهت تحلیل داده‌ها از نرم افزار Instate 3 و آزمون آماری one-way ANOVA استفاده گردید. یافته‌ها: نتایج نشان داد که زهر زنبور عسل در غلطت‌های غیرسمی(5/2 میکروگرم در میلی لیتر) می‌تواند توان تمایزی ویتامین E را افزایش دهد. بحث و نتیجه‌گیری: با توجه به اینکه زهر زنبور به تنهایی بر روی سلول‌هایHL-60 دارای اثر تمایزی نیست، مقایسه اثر تمایزی ویتامین E به تنهایی و در ترکیب با زهر زنبور بر روی سلول‌های HL-60 نشان داد که زهر زنبور می‌تواند همراه با ویتامین E در پیشبرد اثر تمایزی آن موثر بوده و توان تمایزی آن را افزایش دهد. امید است در آینده بتوان از این ترکیب جهت افزایش توان تمایزی ترکیبات تمایز دهنده استفاده نمود.
